# Editorial: AI's transformative role in neuro-intervention: enhancing diagnosis and treatment strategies

**DOI:** 10.3389/fneur.2025.1627547

**Published:** 2025-06-20

**Authors:** Shencai Chen, Donghao Zhang, Fouzi Bala, Hulin Kuang, Aravind Ganesh, Wu Qiu

**Affiliations:** ^1^Department of Neurology, Wuhan Union Hospital, Tongji Medical College, Huazhong University of Science and Technology, Wuhan, China; ^2^School of Life Science and Technology, Huazhong University of Science and Technology, Wuhan, China; ^3^Diagnostic and Interventional Neuroradiology Department, University Hospital of Tours, Tours, France; ^4^School of Computer Science and Engineering, Central South University, Changsha, China; ^5^Department of Clinical Neurosciences, Cumming School of Medicine, University of Calgary, Calgary, AB, Canada

**Keywords:** artificial intelligence, neuro-intervention, precision neurological care, neuroimaging data analysis, neurological disorders

Artificial Intelligence (AI) is poised to revolutionize neuro-intervention and the management of neurological diseases, serving as a transformative force in diagnosis, treatment, and research. By leveraging advanced computational algorithms, AI is reshaping the landscape of neurological care, particularly through its unparalleled ability to analyze complex medical imaging data. This capability enables clinicians to identify subtle patterns and abnormalities that the human eye might miss, leading to faster and more accurate diagnoses. Furthermore, AI-driven tools are increasingly optimizing personalized treatment strategies, allowing the integration of patient-specific factors and real-time data into decision-making processes.

Recent studies have highlighted the substantial impact of AI on clinical practice. For example, machine learning models have demonstrated superior accuracy in detecting early signs of stroke, significantly reducing diagnostic delays (1–3). The development of AI-powered decision-support systems has enabled the tailoring of therapeutic regimens to individual patients, thereby maximizing efficacy and minimizing adverse effects (1, 4). These advancements underscore the growing potential of AI to enhance both the precision and efficiency of neurological care. Despite these achievements, several challenges remain that must be addressed to fully realize AI's benefits. First among these is ensuring the reliability and generalizability of AI models across diverse populations, as models trained on limited or homogeneous datasets may not perform consistently in broader clinical settings (5). In addition, ethical concerns surrounding data privacy, informed consent, and algorithmic transparency are becoming increasingly prominent as AI systems become more integrated into clinical workflows (6). The integration of AI into existing practices also requires overcoming significant technical and logistical barriers, including ensuring interoperability with legacy systems and providing clinician training (7).

This Research Topic highlights recent advances in applying AI to neuro-intervention and nursing. A meta-analysis of 11 RCTs by He et al. found that virtual reality significantly improves motor function, balance, and walking in critically ill patients, though it offers limited gains in functional independence. Deep learning models have shown strong performance in histopathological grading of meningiomas across multiple studies, despite some result heterogeneity, as stated by Noori Mirtaheri et al.. A multi-task learning framework proposed by Nguyen et al. improved predictions of post-stroke health outcomes, outperforming single-task and conventional approaches. Another study by Cao et al. developed an interpretable machine learning model to predict VAP risk in stroke ICU patients, with strong internal validation and enhanced interpretability via SHAP, although generalizability remains a concern. In the research by Teichmann et al., an AI tool for automated segmentation of ischemic stroke lesions showed good agreement with expert annotations, supporting its potential in treatment planning. Overall, while AI shows promise in diagnosis, risk prediction, and rehabilitation, widespread clinical adoption requires further high-quality, large-scale validation.

Looking ahead, Artificial Intelligence and related digital technologies such as virtual reality are poised to transform neuro-intervention and neurological care by enhancing diagnostic accuracy, personalizing treatment, and improving patient outcomes. To realize this potential, future research should focus on refining AI algorithms, expanding their role in prevention and novel therapies, and ensuring their seamless integration into clinical workflows. Collaboration between AI systems and human experts is essential to balance technological innovation with clinical judgment and patient-centered care. However, significant challenges remain, including issues of model generalizability, data privacy, ethical oversight, and multidisciplinary adoption. Addressing these will require rigorous, large-scale validation and sustained collaboration among clinicians, researchers, and technologists to ensure the safe, effective, and responsible clinical implementation of these new tools.
